# Institution for Imbeciles and Idiots

**Published:** 1849-08

**Authors:** H. B. Wilbur

**Affiliations:** Barre, Mass.


We insert the following announcement in consideration of the novelty and interest of the enterprise, which, we trust, may prove successful:—

*Institution for Imbeciles and Idiots.*—This Institution is designed for the management and education of Imbeciles and Idiots.

The system of instruction pursued is of a most comprehensive character, and applicable to every variety of subjects, from the idiot of the lowest grade, up to those with a mere retarded development of mind or will.

It embraces a proper physical education: the education of the senses: the development of functions and aptitudes: the ordinary mental exercises of childhood: and finally the moral treatment or management, including everything calculated to break up the dominion of perverted instincts and animal appetites, and to substitute an intelligent self-control.

The special means adopted are those that have been so successfully employed in the various European Institutions, for the same purpose, and they will severally have that prominence required by each individual subjects. Proper gymnastics, for the full development of the muscular system, and which at the same time serve to cultivate the attentiveness of the pupil. Exercises in imitation, where that faculty is deficient. Sensorial gymnastics; for the education of the senses—of taste and smell—of hearing—of speech—of sight, including all the visible properties of bodies and essential to the formation of correct notions of surrounding objects.

Exercises in drawing. Instruction in writing and reading. The inculcation of notions and ideas. Cultivation of the memory and of will. The various exercises thus summarily enumerated are calculated to awaken dormant functions, to strengthen feeble faculties, and to direct those functions and aptitudes, thus aroused and improved, in the natural channels of physical and mental labor; "to give to the idiot and imbecile, the greatest possible to a child well endowed and properly educated."

But this system does not stop at the mere education of the force, dexterity and intelligence of the pupil. It seeks by the constant and persevering use of every variety of moral means, to render those newly-acquired powers and faculties subservient to an enlightened sense of relations to the moral world.

The success of such a method of instruction upon this unfortunate class is, now, no more questionable than the benefits of the appropriate instruction of the Deaf-mute, or the blind; and the results are even still more surprising. In the one case, the intellect of the pupil is brought to perceive, through the medium of a limited number of senses, educated and exalted, those relations of the external world naturally appreciated by the exercise of all.

In the other, the mind of the subject is released from its isolation, by the obviating of an infirmity of the nervous masses, or of the sensorial apparatus, by proper means of excitation.

And while from a want of positive knowledge of the exact condition of the nervous system, upon which the idiocy depends, a radical benefit cannot always be promised; yet such an amount of improvement, as will amply compensate for the trouble and expense involved in the instruction, can always be predicted.

Due attention will be paid to the health of the pupils, by a proper regimen, the use of baths, and suitable medical treatment, when necessary.

The institution is located in one of the most healthy portions of New England, surrounded by attractive scenery, and of easy access from New York and Boston.

The terms will vary according to the condition of each individual subject, and can be learned by application to the Principal. Persons making applications will please state particulars of the age, sex, health, general form, habits, condition of the senses, speech, mental condition, disposition, and probable cause of the infirmity of the individual for whom the application is made.

The Principal, from his experience in the instruction of imbeciles and idiots, his past success, and his earnest endeavors for the improvement of his pupils, hopes to receive the patronage of those who have children of that class.

H. B. WILBUR, M. D.

*Barre, Mass., May* 1849.

He is kindly permitted to refer to Dr. Samuel B. Woodward, Northampton, Mass., late superintendent of Mass. Insane Hospital.

Dr. Andrew McFarland, superintendent of N. H. Asylum for Insane, Concord, N. H.

Fred. F. Backus, M. D., and Dr. Horatio N. Fenn, Rochester, N. Y.

Prof. Chas. D. Meigs, M. D., and Rev. Samuel A. Clark, Philadelphia.

Rev. J. Addison Cary, institution for the Deaf and Dumb, N. Y. City.

Jeremiah Wilbur, Esq., and Wm. G. Wood, Esq., N. Y. City.

M. S. Perry, M. D., and Mr. J. C. Richards, Boston.

